# Using a Tailored Digital Health Intervention for Family Communication and Cascade Genetic Testing in Swiss and Korean Families With Hereditary Breast and Ovarian Cancer: Protocol for the DIALOGUE Study

**DOI:** 10.2196/26264

**Published:** 2021-06-11

**Authors:** Sue Kim, Monica Aceti, Vasiliki Baroutsou, Nicole Bürki, Maria Caiata-Zufferey, Marco Cattaneo, Pierre O Chappuis, Florina M Ciorba, Rossella Graffeo-Galbiati, Viola Heinzelmann-Schwarz, Joon Jeong, MiSook M Jung, Sung-Won Kim, Jisun Kim, Myong Cheol Lim, Chang Ming, Christian Monnerat, Hyung Seok Park, Sang Hyung Park, Carla A Pedrazzani, Manuela Rabaglio, Jai Min Ryu, Ramon Saccilotto, Simon Wieser, Ursina Zürrer-Härdi, Maria C Katapodi

**Affiliations:** 1 Mo-Im Kim Nursing Research Institute College of Nursing Yonsei University Seoul Republic of Korea; 2 Department of Clinical Research University of Basel Basel Switzerland; 3 Women's Clinic and Gynecological Oncology University Hospital Basel University of Basel Basel Switzerland; 4 La Scuola Universitaria Professionale della Svizzera Italiana (SUPSI) Manno Switzerland; 5 Unit of Oncogenetics, Division of Oncology Division of Genetic Medicine Geneva University Hospitals Geneva Switzerland; 6 Department of Mathematics and Computer Science University of Basel Basel Switzerland; 7 Oncology Institute of Southern Switzerland (IOSI) Bellinzona Switzerland; 8 Department of Surgery Gangnam Severance Hospital, College of Medicine Yonsei University Seoul Republic of Korea; 9 College of Nursing Chungnam National University Deajeon Republic of Korea; 10 Dairim St Mary's Hospital Seoul Republic of Korea; 11 Department of Surgery Asan Medical Center, College of Medicine University of Ulsan Seoul Republic of Korea; 12 Division of Tumor Immunology Center for Gynecologic Cancer Research Institute and Hospital National Cancer Center Goyang Republic of Korea; 13 Oncology Department Jura Hospital Delemont Switzerland; 14 Department of Computer Science Yonsei University Seoul Republic of Korea; 15 University Clinic for Medical Oncology Inselspital Bern Switzerland; 16 Department of Surgery Samsung Medical Center, School of Medicine Sungkyunkwan University Seoul Republic of Korea; 17 School of Management and Law Winterthur Institute of Health Economics Zurich University of Applied Sciences Winterthur Switzerland; 18 Medical Oncology and Hematology Kantonsspital Winterthur Winterthur Switzerland

**Keywords:** HBOC, proportion of informed at-risk relatives, coping, communicating, decisional conflict, cultural and linguistic adaptation, implementation, RE-AIM, mobile phone

## Abstract

**Background:**

In hereditary breast and ovarian cancer (HBOC), family communication of genetic test results is essential for cascade genetic screening, that is, identifying and testing blood relatives of known mutation carriers to determine whether they also carry the pathogenic variant, and to propose preventive and clinical management options. However, up to 50% of blood relatives are unaware of relevant genetic information, suggesting that potential benefits of genetic testing are not communicated effectively within family networks. Technology can facilitate communication and genetic education within HBOC families.

**Objective:**

The aims of this study are to develop the K-CASCADE (Korean–Cancer Predisposition Cascade Genetic Testing) cohort in Korea by expanding an infrastructure developed by the CASCADE (Cancer Predisposition Cascade Genetic Testing) Consortium in Switzerland; develop a digital health intervention to support the communication of cancer predisposition for Swiss and Korean HBOC families, based on linguistic and cultural adaptation of the Family Gene Toolkit; evaluate its efficacy on primary (family communication of genetic results and cascade testing) and secondary (psychological distress, genetic literacy, active coping, and decision making) outcomes; and explore its translatability using the reach, effectiveness, adoption, implementation, and maintenance framework.

**Methods:**

The digital health intervention will be available in French, German, Italian, Korean, and English and can be accessed via the web, mobile phone, or tablet (ie, device-agnostic). K-CASCADE cohort of Korean HBOC mutation carriers and relatives will be based on the CASCADE infrastructure. Narrative data collected through individual interviews or mini focus groups from 20 to 24 HBOC family members per linguistic region and 6-10 health care providers involved in genetic services will identify the local cultures and context, and inform the content of the tailored messages. The efficacy of the digital health intervention against a comparison website will be assessed in a randomized trial with 104 HBOC mutation carriers (52 in each study arm). The translatability of the digital health intervention will be assessed using survey data collected from HBOC families and health care providers.

**Results:**

Funding was received in October 2019. It is projected that data collection will be completed by January 2023 and results will be published in fall 2023.

**Conclusions:**

This study addresses the continuum of translational research, from developing an international research infrastructure and adapting an existing digital health intervention to testing its efficacy in a randomized controlled trial and exploring its translatability using an established framework. Adapting existing interventions, rather than developing new ones, takes advantage of previous valid experiences without duplicating efforts. Culturally sensitive web-based interventions that enhance family communication and understanding of genetic cancer risk are timely. This collaboration creates a research infrastructure between Switzerland and Korea that can be scaled up to cover other hereditary cancer syndromes.

**Trial Registration:**

ClinicalTrials.gov NCT04214210; https://clinicaltrials.gov/ct2/show/NCT04214210 and CRiS KCT0005643; https://cris.nih.go.kr/cris/

**International Registered Report Identifier (IRRID):**

PRR1-10.2196/26264

## Introduction

### Background

In 2018, there were approximately 2.1 million breast cancer diagnoses and more than 600,000 associated deaths worldwide [1,2]. The worldwide average breast cancer incidence is 74.2 per 100,000 women [3], with approximately 25% of cases occurring in women younger than 50 years and in women with a family history of cancer [4,5]. Approximately 5%-10% of breast cancer and 20% of ovarian cancer cases occur due to germline pathogenic variants associated with hereditary breast and ovarian cancer (HBOC) syndrome, most commonly observed in the *BRCA-1* and *BRCA-2* genes (hereafter *BRCA*). The prevalence of germline pathogenic variants differs among ethnic groups [6]; however, Switzerland and Korea have a similar prevalence ranging from 23% to 26% [7,8].

The availability of genetic services (counseling and testing) for actionable hereditary cancer syndromes such as HBOC enables population-level cancer prevention [9]. Blood relatives of HBOC cases have a 12.5%-50% probability of inheriting the same pathogenic variant and can be tested with 100% accuracy. Chemoprevention, prophylactic surgery, and intensive surveillance can lower cancer risks for relatives who test positive, whereas those who test negative are excluded from these interventions [10-12]. The Centers for Disease Control and Prevention Office for Public Health Genomics recommend genetic testing in cancer-free individuals with a known HBOC family history and in patients with cancer who have strong indications of HBOC syndrome (eg, ovarian cancer) [13]. Cascade genetic screening is a systematic effort to identify and test all blood relatives of HBOC cases to determine whether they also carry the same pathogenic variant [10]. The CASCADE (Cancer Predisposition Cascade Genetic Testing) Consortium in Switzerland promotes cascade genetic screening for HBOC [14,15], whereas the Korean Hereditary Breast Cancer (KOHBRA) network identifies the prevalence of HBOC-associated pathogenic variants in the Korean population [16,17].

Despite calls to action for HBOC cascade genetic testing, there are systemic barriers to its implementation. Privacy laws worldwide, including Switzerland and Korea, restrict health care providers from revealing genetic information to anyone except the tested individual, who has the right not to disclose this information, despite implications for relatives’ health [18-20]. The potential benefits of genetic testing are not being effectively communicated through family networks, leading to more than 50% of at-risk individuals not using genetic services and not receiving important information from a credible source [21-23]. Second-degree and male relatives, those living further away, and those with an estranged relationship with the mutation carrier are most likely not to be informed about genetic testing [24,25]. Despite these difficulties, a family-based approach in communicating hereditary cancer risk is advantageous because it may reach relatives through the social bonds and functions already existing within the family network, and it is not limited to those in contact with the health care system [26].

Interventions that support mutation carriers during the disclosure of genetic test results can reduce psychological distress and provide relatives with accurate and credible information about cascade genetic testing. Technology-enabled education is not inferior to face-to-face genetic consultations [27,28], but it increases access to services and cost-effectiveness [29-32]. The Family Gene Toolkit [33] is a web-based intervention designed to increase prerequisites for HBOC cascade testing, that is, active coping, open family communication, and informed decision making. The prototype Family Gene Toolkit was tested in the United States for acceptability and patient satisfaction with excellent results, confirming its value for these families. However, it is not available in other linguistic and cultural contexts. Adapting existing interventions, rather than developing new ones, takes advantage of the previous valid experiences without duplicating efforts.

In summary, HBOC cascade genetic testing, meaning the identification and testing of blood relatives, provides risk management options for those with a germline pathogenic variant and excludes confirmed noncarriers (ie, negative testing when there is a known pathogenic variant in the family) from intensive surveillance and risk-reducing measures. Due to privacy laws, mutation carriers have the sole responsibility to inform blood relatives about genetic test results and advocate for genetic services. Digital health platforms can support mutation carriers during the disclosure process and provide relatives with accurate and credible information.

### Objectives

The DIALOGUE study will build a bilateral research infrastructure to support collaboration and multidisciplinary initiatives around HBOC in Switzerland and Korea. The specific aims are to develop the K-CASCADE (Korean–Cancer Predisposition Cascade Genetic Testing) cohort in Korea by expanding an existing research infrastructure developed by the CASCADE Consortium in Switzerland; develop a digital health intervention to support open communication and cascade genetic testing in HBOC families, based on the linguistic and cultural adaptation of the Family Gene Toolkit; evaluate the efficacy of the digital health intervention on primary (communication of genetic test results to relatives and cascade genetic testing) and secondary (psychological distress, genetic literacy, coping, and decision making) outcomes; and explore the translatability of the platform using the reach, effectiveness, adoption, implementation, and maintenance (RE-AIM) framework.

## Methods

### Design

The DIALOGUE study will use a cohort design to establish the K-CASCADE in Korea and a randomized controlled trial (RCT) design to test the effects of digital health intervention in the Swiss and Korean contexts. The study will measure clinical and process outcomes in real-world conditions, including different settings, participants, and resources [34,35].

#### Aim 1: Develop the K-CASCADE Cohort

The K-CASCADE cohort will identify and survey mutation carriers and blood relatives as its archetype, the Swiss CASCADE cohort, using similar design, assessments, and procedures for sample identification and data collection [14]. Adult Korean men and women with *BRCA* pathogenic variants will be invited to the K-CASCADE cohort. They will also be asked to invite their first- and second-degree relatives and their first cousins for cascade genetic screening. Similar to the Swiss CASCADE, it is envisioned that the K-CASCADE cohort will include known mutation carriers, untested relatives with unknown mutation status, and relatives who tested negative for the pathogenic variant.

#### Aim 2: Adapt the Digital Health Intervention

**Figure 1:**
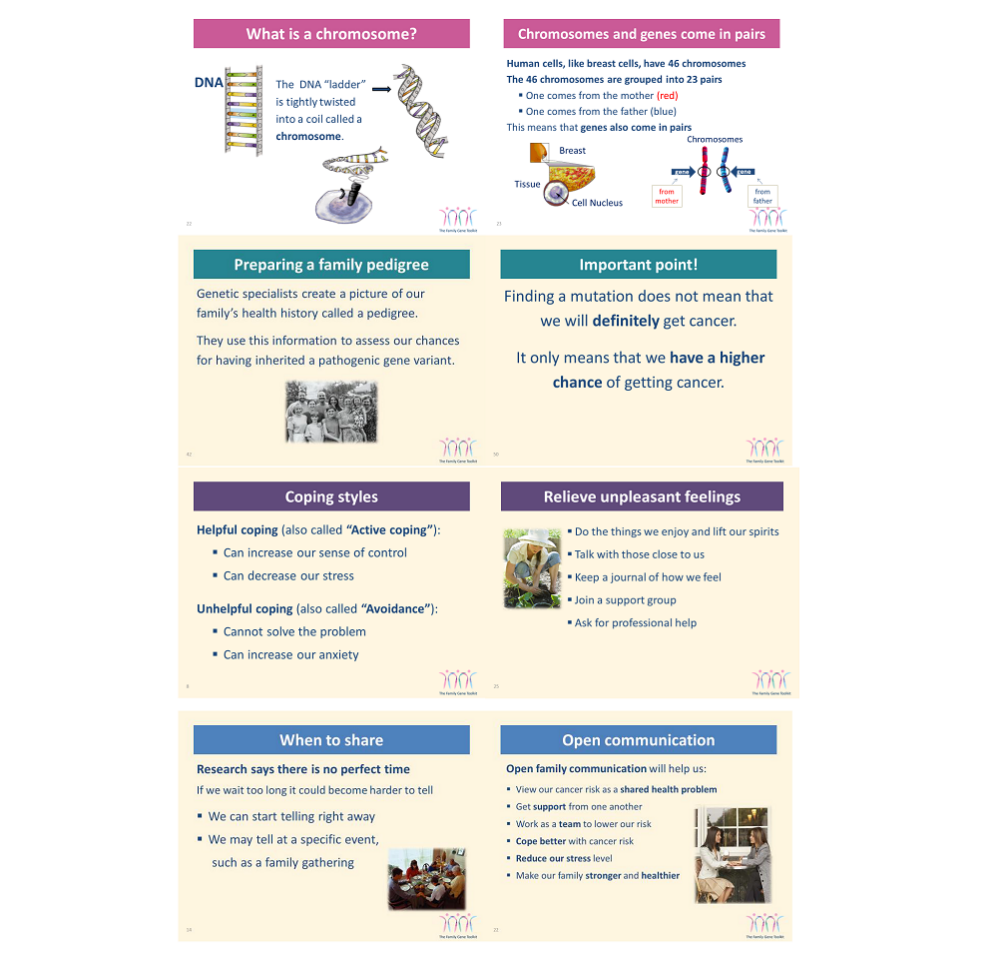
Examples from the Family Gene Toolkit.

The content of the Family Gene Toolkit is driven by theory [36] and supported by empirical findings [37-41]. It is designed to address challenges related to the quantity and complexity of genetic information mutation carriers are asked to communicate with family members [42,43]. Understanding HBOC (eg, probability of mutation, prognosis, prevention, and treatment) and the accuracy of genetic testing are important for decision making. Inherited cancer risk requires ongoing management and, thus, active coping with health challenges. Mutation carriers’ personal values and communication skills are important for the disclosure of genetic cancer risk. The Family Gene Toolkit embraced the above challenges and included 4 modules designed to increase knowledge of cancer genetics (module 1), provide decisional support for genetic testing to untested relatives (module 2), increase active coping with common challenges faced by HBOC families (module 3), and provide skills-building communication training (module 4; Figure 1). The adapted Family Gene Toolkit will include the 4 original modules and a fifth module about the management of cancer risk based on recommendations from the National Comprehensive Cancer Network [44].

The research team will create tailored messages based on the linguistic and cultural adaptations of the modules. Tailoring is a process that *fits* the message to meet one’s personal needs and characteristics, rather than targeting group criteria [45,46]. Tailored messages improve whether and how one listens to a message and its impact on behavior change. Shallow tailoring involves elements of appearance (eg, female or male mutation carriers), whereas deep tailoring involves more complex elements of relevance (eg, coping style). Adaptation of the Family Gene Toolkit involves elements of both shallow and deep tailoring based on preintervention assessments of participants’ characteristics such as sex, affected with cancer versus cancer-free, and tendency to rely more on a specific coping style. The research team will use readily available e-learning products with different tailored messages, multiple interactions and assessments, and a device-agnostic interface for the adaptation of the Family Gene Toolkit. Messages will be developed in English and translated at the eighth-grade reading level while considering Swiss and Korean legislation, health insurance policy, cultural values, and national languages. Swiss and Korean stakeholders will review the content of the adapted Family Gene Toolkit and identify the required modifications by providing feedback on word choices, sensitivity of messages, and appearance. Mini focus groups and individual interviews with clinicians involved in genetic consultations will evaluate the prototype of each module and the tailoring elements. Focus groups with Swiss and Korean HBOC mutation carriers and relatives will provide suggestions to enhance the comprehensibility, usefulness, acceptability, and feasibility of the intervention. Feedback from clinicians and HBOC families will help in further refining each module and the tailored messages.

Assessing the usability of the adapted Family Gene Toolkit involves task-oriented assignments about the most important functions and features of the website as well as the ease and user-friendliness of navigation. Participants will *think aloud* while navigating each module and complete each task [47]. They will also evaluate the tailored messages for readability and comprehension.

Swiss and Korean participants will complete the 5 modules at their own pace, but within a timeframe of 4 weeks after they first engage with the platform. The 4-week interval enables information assimilation and adequate time to reflect and act based on tailored messages while providing a controlled learning environment. Feedback will be based on baseline responses, including tailored advice about improvements that can be made.

Consistent with testing real-world alternatives [48], the DIALOGUE study will provide a comparison website with targeted (generic) information. The comparison website will mimic the structure and functions of an existing website [49]. The adapted Family Gene Toolkit and the comparison website will be technically implemented in the same system that will collect baseline and follow-up data, randomize participants, deliver the intervention and the comparison website, track access and use of the platform, and provide a user-friendly experience to participants.

#### Aim 3: Evaluate Intervention Efficacy on Primary and Secondary Outcomes

A cluster RCT will evaluate the magnitude of intervention effects as compared with the comparison website. Randomization will occur at the family level, that is, after baseline data collection, the digital health intervention will randomly assign mutation carriers to either intervention arm, stratifying for country. Invited relatives will be automatically directed to the same arm as the mutation carrier. All study participants will complete a survey at baseline (T_1_) before the intervention and again at 2 months (T_2_) and 6 months (T_3_) after the intervention. The 2- and 6-month follow-up time points will assess the short-term and long-term effects in line with our previous studies [33,50].

#### Aim 4: Explore Intervention Translatability

The implementation and dissemination of the adapted Family Gene Toolkit will be evaluated based on the constructs of the RE-AIM framework [51] at the individual and organizational levels.

### Settings

The DIALOGUE study involves oncology and genetic testing centers of the Swiss CASCADE Consortium from 3 linguistic regions of Switzerland (German-, French-, and Italian-speaking) and similar settings in Korea, eg, Severance Hospital, Seoul, and the National Cancer Center, Goyang. Settings ensure diversity in hospital characteristics (eg, general or advanced level) and geographic location to increase sample representativeness and generalizability.

### Sample and Sample Size

The DIALOGUE study targets individuals who have been identified through genetic testing as carrying a *BRCA* pathogenic variant (proband) and their blood relatives. Textbox 1 describes the inclusion criteria for the probands and relatives. Eligible probands will be females and males (expected female-to-male ratio=4:1) and their first- and second-degree relatives (parents, siblings, offspring, aunts, uncles, nieces, nephews, and grandparents) and their first cousins. Participants may have a cancer diagnosis (expected breast-to-ovarian cancer ratio=5:1) or they may be cancer-free. Individuals who tested positive for a variant of uncertain significance and mutation carriers without any blood relatives, spouses, and partners are excluded because cascade genetic testing does not apply to them. We also exclude individuals who tested positive for a non-*BRCA* pathogenic variant because of the current variation in the implementation of panel testing among the different sites, which will likely influence the recruitment of participants with non-*BRCA* mutations. The study will only include adults because hereditary cancer risk assessment is not recommended for children. The study will also exclude vulnerable participants, such as critically ill patients and those living in nursing homes, to avoid increasing the subject burden and provide surveillance recommendations to participants who are not able to follow through the program.

**Inclusion criteria.**

Switzerland

- Probands
  Has tested positive for a BRCA pathogenic variant
  ≥1 first- or second-degree relative or first cousin
- Probands and relatives
  ≥18 years old
  German, French, Italian, and English
  Swiss resident
  Mentally able to provide written consent
  Access to computer, tablet, or smartphone

Korea

- Probands
  Has tested positive for a BRCA pathogenic variant
  ≥1 first- or second-degree relative or first cousin
- Probands and relatives
  ≥19 years old
  Korean and English
  Korean resident
  Mentally able to provide written consent
  Access to computer, tablet, or smartphone

#### K-CASCADE Cohort

Estimates of sample accessibility follow consultations with the medical directors of clinical sites and assume average HBOC prevalence rates of 5% for both countries. There are approximately 90 new mutation carriers per year from the clinical centers affiliated with the Swiss CASCADE Consortium. Data from the Swiss CASCADE cohort indicate that it is feasible to recruit approximately 50% of the probands from each clinical site. Each proband is willing to invite an average of 4 relatives, with a response rate of 50% among relatives. In Korea, with 5 participating hospitals, 540 individuals are expected to enter the K-CASCADE cohort over 3 years (6 individuals per month×12 months×3 years×5 hospitals, 50% participation rate).

#### Intervention Adaptation

A purposeful sample of 20-24 participants (10-12 mutation carriers and 10-12 relatives) per linguistic region will participate in individual interviews and/or mini focus groups in each country. There will be homogeneity within members of each focus group, but the samples will be diversified in terms of demographics (eg, sex) and clinical history (eg, affected with cancer vs cancer-free) among groups. Mini focus groups or interviews will be conducted with a convenience sample of 6-10 expert clinicians involved in genetic consultation in each country. Mini focus groups allow more time to share experiences and encourage greater in-depth information and insights. The adapted Family Gene Toolkit will be tested for usability and acceptability with 5 new mutation carriers and/or relatives per linguistic region [52,53].

#### Intervention Efficacy

The cluster RCT will invite 114 probands to have a total of 104 evaluable subjects (52 for each website). This sample size would allow detecting whether using the adapted Family Gene Toolkit or the comparison website would increase the proportion of informed relatives by 25% with a statistical power of 80%, a significance level of 5%, and a dropout rate of 9.6% (11/114). We estimated the distribution of the proportion of relatives and dropout rates from our previous studies [14,50]. Less than 10.5% (12/114) of potential participants have no relatives, and less than 10.5% (12/114) have no access to a computer, tablet, or smartphone, making them ineligible for the study. We expect to recruit and retain the final sample of 114 probands over 18 months.

#### Intervention Translatability

This step involves assessing the potential for implementing the adapted Family Gene Toolkit in real-world conditions. RE-AIM dimensions will be assessed from participating and nonparticipating mutation carriers, relatives, and providers throughout the study.

### Recruitment and Procedures

#### K-CASCADE Cohort

The opportunity to participate in the K-CASCADE cohort will be advertised through the KOHBRA network, the Ovarian Cancer and Genetics study group, and other clinical sites. Recruitment procedures for Korean probands and relatives will follow steps and procedures similar to those outlined for the Swiss CASCADE cohort [14]. In short, index cases (first person in the family with the pathogenic variant) identified in participating centers will be invited to participate in the study by collaborating clinicians and through patient advertisements posted in the clinics. Potential participants will also be able to view information on the study website. Individuals carrying a *BRCA* pathogenic variant, and if they have at least one eligible relative based on pedigree data, will meet study recruiters to ask questions and provide written consent. To alleviate ethical concerns associated with contacting blood relatives without their explicit consent, the K-CASCADE cohort will approach them through probands, targeting only relatives the proband is willing to contact. This recruitment method is used by the Swiss CASCADE cohort and in previous family-based studies with very good recruitment outcomes [39,54]. Relatives agreeing to participate will also provide written consent. In the consent form, probands and relatives will indicate their willingness to invite additional relatives to the K-CASCADE, be contacted once a year for 5 years and provide updated information about their health, participate in a focus group or individual interview for the adaptation of the Family Gene Toolkit in Korean, and participate in an RCT for testing the effects of the adapted Family Gene Toolkit. Probands and relatives may participate in all or some of the study steps previously described.

Probands and relatives will provide baseline assessments via a URL link to the digital health intervention and a unique passcode. A second prompt will be sent 2 weeks later. If there is no response to the second contact, study recruiters will contact the participants by phone. Relatives will also provide written consent, and they will receive a URL with a unique passcode. The Swiss CASCADE platform will facilitate data collection in both countries to maintain the consistency and accuracy of data entry, data management, and analyses. Korean respondents will log on as K-CASCADE participants to provide survey data.

#### Intervention Adaptation

Participants will be recruited through the Swiss CASCADE cohort and through flyers posted in the affiliated Korean institutions and clinics. After obtaining consent, focus groups or individual interviews will be organized at an easily accessible site and in participants’ language. Focus groups will be coded by 2 members of the team in each country and linguistic region and will be audiotaped with participants’ explicit consent. Participants will be asked to *think aloud* while viewing electronic mockups of the intervention and while navigating a final version of the digital health intervention. The latter sessions will be videotaped.

Clinicians involved in genetic consultations will be identified through the CASCADE Consortium in Switzerland; through the Schweizerischen Arbeitsgemeinschaft für Klinische Krebsforschung, Network for Cancer Predisposition Testing and Counseling in Switzerland; and through the KOHBRA network in Korea. They will be recruited via email and/or invitation letters and will also provide consent. Semistructured exploratory questions will elicit their opinions on structural barriers to HBOC cascade genetic testing. At a later stage, they will also view a nearly final version of the digital health intervention and will provide feedback. Sessions will be audiotaped and videotaped with clinicians’ consent.

#### Intervention Efficacy

After they complete the baseline questionnaires (T_1_), probands (index case) in both countries who agree to participate in an RCT and test the effects of the adapted Family Gene Toolkit will be emailed a unique URL link and passcode allowing them access to the digital health intervention. Furthermore, they will be able to log in and review the intervention modules multiple times using the same URL link and passcode. The system will randomize participants in a 1:1 ratio to either the digital health intervention or the comparison website. Stratification by country (Switzerland vs Korea) will be facilitated with different URL links for participants from each country. Participants will receive weekly email or text alerts, encouraging them to visit the website and complete viewing of the contents of the digital health intervention within 4 weeks. They will also receive email or text alerts to complete a knowledge quiz, an exercise for value clarification related to genetic testing, and a family communication rubric that will be included in the content of the different modules. Participants randomized to the comparison website will receive 1 email alert 2 weeks after they engage with the website. Relatives will be allocated to the same study arm as the respective proband and will also receive a URL link and a unique passcode. Relatives will first be asked to complete a consent form and then to complete the baseline survey, after which they will have access to either the adapted Family Gene Toolkit or the comparison.

Primary and secondary outcomes will be assessed at 2 months (T_2_) and 6 months (T_3_) after the intervention. We selected the 2- and 6-month follow-up time points to measure the short-term and long-term intervention effects, in line with our previous studies [33,50]. To minimize the attrition rate, if a response has not been received within 2 weeks from the time participants receive the URL link to the follow-up survey, then the study personnel will make 3 attempts to contact them by email or phone and encourage them to complete the survey.

### Measures and Outcomes

#### K-CASCADE

The core questions of the Swiss CASCADE cohort [14] constitute the basic measurements for the K-CASCADE. Instruments are purchased (if not available for free) and will be translated into Korean (if not available) following the World Health Organization’s translation guidelines. The baseline survey covers cancer diagnoses and surveillance, use of and experience with genetic testing (for testers and nontesters), communication with health care providers, and satisfaction with cancer genetic services. It assesses information about prophylactic surgeries; epidemiological data about personal, reproductive, and family history of breast and ovarian cancer; and modifiable lifestyle risk factors (smoking, drinking, physical activity, etc). The baseline survey also assesses demographic characteristics and psychosocial variables, for example, the fear of cancer recurrence and self-efficacy to use services, which constitute the basis for creating the tailored messages provided by the adapted Family Gene Toolkit. These instruments are listed in Table 1. The Korean survey will be pilot tested with 10 study participants for comprehension and accuracy.

**Table 1:** Demographics and psychosocial characteristics.

Variables			Instruments		Cronbach α		Test-retest reliability		Assessment		
									Baseline		Follow-up
Demographics, personal, and family cancer history			Self-report [55]		—^a^		—		✓^b^		
**Tailoring variables**											
	Degree of relationship between proband-relatives (eg, first degree)	Self-report		N/A^c^		N/A		✓			
	Fear of cancer recurrence (for patients)	Concerns About Recurrence Scale [56], 4 items, 7-point Likert scale		.93		0.91		✓			
	Self-efficacy dealing with cancer (for patients)	Self-efficacy–HBOC^d^-related cancer [57], 14 items, 7-point Likert scale		.80		0.71		✓			
	Self-efficacy using genetic services	1 item, 7-point Likert scale		N/A		N/A		✓			
	Family support	Family Support in Illness [58], 10 items, 7-point Likert scale		.86		0.83		✓			
	Family hardiness	Family Hardness Index [59], 20 items, 7-point Likert scale		.90		0.82		✓			
	Satisfaction with genetic counseling (for tested individuals)	Multidimensional Impact of Cancer Risk Assessment [60], 19 items, 7-point Likert scale		.81		—		✓			
	Barriers and facilitators for genetic services	Barriers and facilitators for genetic services [61], 11 items, multiple choice		N/A		N/A		✓			

^a^Not available.

^b^The variable will be assessed at the specific time frame.

^c^N/A: not applicable.

^d^HBOC: hereditary breast and ovarian cancer.

#### Intervention Adaptation

A trained moderator will ask focus group participants to answer semistructured exploratory questions designed to elicit their opinions on the most pressing issues for family communication, using appropriate probe questions to explore potential cultural interpretations. The interview guide explores issues around family communication that took place during the genetic consultation, decision making related to the disclosure of test results to relatives, and attitudes toward using digital health platforms. Participants will also rate their satisfaction with the content, format, and appearance of the website. Assessing intervention feasibility also involves assessing the number of modules accessed, time spent on each module, and the utilization of links, which are automatically recorded on the website.

#### Intervention Efficacy

Data to evaluate the magnitude of intervention effects will be assessed using the instruments listed in Table 2. These have strong psychometric properties and have been used in previous studies on patients with cancer. Most of these instruments have been translated into and validated in German, French, and Italian and will be translated into and validated in Korean. Primary and secondary outcomes are assessed at the 2-month and 6-month follow-up surveys. Satisfaction with the intervention and acceptability will be assessed at the 2-month follow-up with questions about intervention usefulness, ease of use, clarity, appropriate length, level of detail, relevance, and interest with a 7-item survey (Likert scale ranging from 1=low to 7=high) [62,63].

**Table 2:** Instruments to assess primary and secondary outcomes.

Concepts			Instruments		Cronbach α		Test-retest reliability		Assessment		
									Baseline		Follow-up
**Primary outcomes**											
	Proportion of informed relatives	Website data		N/A^a^		N/A		✓^b^		✓	
	Intention to inform relatives	Informing Relatives Inventory [64], 68 items, 7-point Likert scale		.86		—^c^		✓		✓	
	Intention for genetic testing (untested relatives)	1 item, 7-point Likert scale		N/A		N/A		✓		✓	
**Secondary outcomes**											
	Psychological distress	Profile of Mood States [65], 37 items, 7-point Likert scale		.86		—		✓		✓	
	Genetic literacy—genetic affinity	Risk Factor Knowledge Index [39], 17 items, true, false, and do not know		.89		0.85		✓		✓	
	Genetic literacy—cancer genetics	Breast Cancer Genetics Index [66], 12 items, true, false, and do not know		.82		0.81		✓		✓	
	Coping with stressful events	Brief Cope [67], 25 items, 7-point Likert scale		.81		0.78		✓		✓	
	Decision making—untested individuals	Decisional Conflict Scale-HBOC^d^ Genetic Testing [68], 16 items, 7-point Likert scale		.96		—		✓		✓	
	Decision making—tested individuals	Decisional Regret-Genetic Testing [69], 5 items, 7-point Likert scale		.87		—		✓		✓	
**Intervention evaluation**											
	Acceptability, detail, usefulness, relevance, and satisfaction	Intervention Evaluation [62,70], 16 items, 7-point Likert scale		—		—				✓	

^a^N/A: not applicable.

^b^The variable will be assessed at the specific time frame.

^c^Not available.

^d^HBOC: hereditary breast and ovarian cancer.

#### Intervention Translatability

Textbox 2 outlines RE-AIM outcomes to be assessed, which will help in evaluating the potential for a broader implementation and dissemination of the digital health intervention.

**Reach, effectiveness, adoption, implementation, and maintenance outcomes assessed in the study.**

Reach (individual)

- Response rate of mutation carriers and relatives
- Number of probands and relatives accessing the website
- Demographic, linguistic characteristics, and region
- Response rate to K-CASCADE (Korean–Cancer Predisposition Cascade Genetic Testing)

Effectiveness (individual)

- Assess the number of times participants accessed each module
- Assess the number of relative invites initiated through the website
- Evaluate the acceptability, interest, usefulness, level of detail, relevance, and satisfaction follow-up survey

Adoption (setting, staff, and organization)

- Number of clinicians and new settings willing to participate in the study
- Diversity (geographic, linguistic, etc) in participating settings

Implementation (setting, staff, and organization)

- Monitor referrals of mutation carriers from different clinical sites
- Evaluate the cost for adapting modules for other hereditary cancer syndromes (eg, Lynch syndrome)

Maintenance (individual and setting)

- Assess resources needed to maintain the website
- Assess the number of visits per month per year

### Data Management and Data Analyses

#### K-CASCADE Cohort

Korean participants’ data entered in the Swiss CASCADE platform will be available for descriptive and comparative analyses, using epidemiological and psychosocial data along with coded and nonidentified clinical data. Existing clinical data from Severance Hospital, stored in the Clinical Research Analysis Portal, will also be accessed for participants who provide additional consent. At year 4, the accrued data from Korean women will be used in conjunction with clinical data for comparative analyses with the Swiss CASCADE cohort.

#### Intervention Adaptation

The mini focus groups or interviews with HBOC families and clinicians will be audiorecorded with participants’ consent and transcribed verbatim, using codes to protect individual identification. Transcripts will be reviewed by the research team, and content will be analyzed using an iterative process of reading transcripts, coding, and comparing the data to identify salient themes. Two members of the research team in each country will also review the videotapes obtained from usability testing and the *think aloud* protocol. They will confirm that there are no functional errors on the website, color schemes and graphical images are well received, participants can navigate through various sections of the website with ease, the layout accurately conveys information, and the program works as expected. Data regarding acceptability will be analyzed using descriptive statistics.

#### Intervention Efficacy

The efficacy cluster RCT will use pre- and postintervention data from baseline (T_1_) and follow-up (T_2_ and T_3_) surveys. Data values will be checked for validity (within the appropriate range) using histograms and box plots and corrected whenever possible. Many items are a part of multi-item scales and are anticipated to correlate with each other. Scales will be tested for internal consistency reliability with Swiss and Korean participants using principal component analysis and Cronbach α coefficients. Scales with Cronbach α values of .71 and higher will be used. Multiple imputation or other techniques will address missing data if they exceed 5% of observations and if they are less than 25% for each specific scale. Data from participants who withdraw will be kept to ensure internal validity.

Primary outcomes will be calculated with the Wilcoxon-Mann-Whitney test to compare the proportions of informed relatives per study arm. Other primary and secondary outcomes and metadata from the automatic recording of website activity will be analyzed using descriptive statistics. Descriptive analyses will include calculating the means and frequencies of key variables and subject descriptors (eg, genetic testing). This will include tabulating counts and frequencies of variables, including demographics and personal cancer history. Bivariate analyses (using the chi-square test for differences in proportions and *t* test for differences in means) will assess the associations between demographic factors and clinical characteristics. The following comparisons will be made: between probands and relatives, between men and women, between patients with cancer and cancer-free individuals, between participants with children and those with no children, between different age groups, and between patients with different cancer diagnoses. A detailed methodology for summaries and statistical analyses will be documented in a statistical analysis plan. This plan will be ﬁnalized before database closure and will be under version control at the Clinical Trial Unit, University Hospital Basel. All analyses will be conducted using the statistical software R [71], using *two-sided* statistical tests and conﬁdence intervals with conﬁdence levels α=5% and (100%−α)=95%, respectively. Deviations from planned analyses are not foreseen. The study statistician will review and approve any deviations from the original statistical plan.

#### Intervention Translatability

Data exploring the RE-AIM of the digital health intervention will be analyzed using qualitative and quantitative methods. Narrative data obtained from mini-interviews will be audiorecorded, transcribed verbatim, and analyzed for common themes. Descriptive analyses will include calculating the means and frequencies of the key variables and subject descriptors. Bivariate analyses (chi-square test for differences in proportions and *t* test for differences in means) will compare key variables between participants and nonparticipants.

## Results

The DIALOGUE study, including the development of the K-CASCADE cohort in Korea, was funded in October 2019. It is projected that data collection will be completed by January 2023, and results will be published in fall 2023.

## Discussion

### Principal Findings

The need to enhance family communication around HBOC has been documented in the literature since mid-2000 [72-74], followed approximately 10 years later by scientific calls to enhance cancer predisposition cascade genetic testing [75-77]. The DIALOGUE study is a resource-effective international research platform that proposes building a tailored, interactive website to reach a large number of HBOC families and enhance cancer predisposition cascade genetic screening, presumably requiring only a fraction of the cost and required clinician time compared with previous approaches. Developing the K-CASCADE cohort will link together the expertise of an eminent network of HBOC scholars and clinicians that will benefit both countries and serve as a model for potential expansion to other countries and in other language contexts. The cross-cultural adaptation of the Family Gene Toolkit will help explore the similarities and differences in communication practices among HBOC families in the Swiss and Korean contexts, potentially providing important information about the Korean and Swiss contexts that affect HBOC discourse [78]. This comparison will also reveal context-specific characteristics regarding the influence of the health care system, insurance coverage, and socioeconomic aspects on the application of genetic knowledge that can provide useful information for adapting other digital health solutions within the Swiss and Korean contexts. The goal of the adapted Family Gene Toolkit is to attend to the needs of diverse families, including the function of different members, and cultural and linguistic backgrounds. It is thus important to consider digital health technologies as sociocultural products with a need for an adaptation to specific local contexts and a critical reflection about how they may affect local perceptions of illness [78]. The final product will likely be more cost-effective and will expedite scaling-up, dissemination, and implementation, given the existing strong clinical partnerships within each country.

### Conclusions

The adaptation and implementation of culturally sensitive, digitally based health interventions that enhance the understanding of genetic cancer risk are extremely timely and relevant, given the expansion of genetic testing technology, the falling costs of genetic testing, and the increased pressure for the integration of genetic knowledge in routine clinical care. Genetic testing for hereditary susceptibility to disease has received increasing attention among the health care community and at the individual, familial, and international levels. The DIALOGUE study will contribute to the development of high-quality comprehensive support systems that enhance the counseling process and facilitate informed decision making by minimizing conflict and distress and making resources available in culturally appropriate ways. Ultimately, the study contributes to a broader dissemination of genetic information and helps in expanding the public health understanding of the impact of new technologies on risk stratification and disease management.

## Abbreviations

CASCADE: Cancer Predisposition Cascade Genetic Testing
HBOC: hereditary breast and ovarian cancer
K-CASCADE: Korean–Cancer Predisposition Cascade Genetic Testing
KOHBRA: Korean Hereditary Breast Cancer
RCT: randomized controlled trial
RE-AIM: reach, effectiveness, adoption, implementation, and maintenance
